# Expression of Von Hippel – Lindau (VHL) gene mutation in diagnosed cases of renal cell carcinoma

**DOI:** 10.12669/pjms.304.4733

**Published:** 2014

**Authors:** Humera Shahzad, Shahnaz Imdad Kehar, Shahzad Ali, Naila Tariq

**Affiliations:** 1 Humera Shahzad, Lecturer, Pathology Department, Basic Medical Sciences Institute, Jinnah Postgraduate Medical Centre (JPMC), Karachi, Pakistan.; 2 Shahnaz Imdad Kehar, Associate Professor, Pathology Department, Basic Medical Sciences Institute, Jinnah Postgraduate Medical Centre (JPMC), Karachi, Pakistan.; 3 Shahzad Ali, Associate Professor, Urology Department, Jinnah Postgraduate Medical Centre (JPMC), Karachi, Pakistan.; 4 Naila Tariq, Assistant Professor, Clinical Pathology Department, Jinnah Postgraduate Medical Centre (JPMC), Karachi, Pakistan.

**Keywords:** Renal cell carcinoma (RCC) Clear cell renal cell carcinoma (CCRCC), Clear cell papillary renal cell carcinoma (CCPRC), Papillary RCC (PRCC), Von Hippel Lindau (VHL) gene

## Abstract

***Objective: ***To evaluate the expression of Von Hippel Lindau (VHL) gene in diagnosed cases of renal cell carcinoma.

***Methods:*** This cross sectional study was conducted in department of Pathology, Basic Medical Sciences Institute, JPMC, Karachi, from January 2007 to December 2012. Paraffin embedded blocks of 30 cases of radical nephrectomy specimens diagnosed as renal cell carcinoma including CCRCC 21 (70%) CCPRCC, 3 (10%), PRCC 2 (6.79%), hybrid tumor 4 (13.3%), chromophobe tumor (0%) processed for VHL gene expression on Polymerase Chain Reaction.

***Results:*** All the 30 cases previously diagnosed as renal cell carcinoma were processed on PCR, VHL gene mutations were seen in 20 (95.23%) of CCRCC while a single case was negative for VHL mutations. All CCPRCC were negative for VHL mutation. Among the hybrid tumor 03 cases with foci of clear cells show VHL mutation while a single case showing combination of clear cells and chromophobe cells was negative for mutation. Both the cases of PRCC were positive for mutation. Exon 3 mutation at base pair 194 seen in 8 (32%) cases and Exon 2 mutation at base pair 150-159 seen in 17 (68%) cases. None of the cases showed Exon 1 mutation.

***Conclusion:*** The present study shows that majority of CCRCC showed VHL mutation including the hybrid tumor with clear cell component in our population.

## INTRODUCTION

Von Hippel Lindau (VHL) is a tumor suppressor gene, located on chromosome 3. A mutation in both copies of VHL gene within the same cell increases the risk of certain neoplasm. VHL carriers have a greatly increased risk of developing tumors compared to the general population. The most characterized form of hereditary renal carcinoma is seen in Von-Hippel Lindau syndrome which is a hereditary autosomal dominant syndrome. In this condition individuals are at risk to develop tumors like hemangiomas found in cerebellum, spinal cord, kidney and retina, angiomas and pheochromocytoma.^1^

A considerable number of VHL gene mutations are not inherited from the parents. Therefore 75% to 80% of renal cell carcinomas are sporadic and show biallelic VHL gene defect. The Clear cell variant of renal cell carcinoma referred to as conventional renal cell carcinoma is the most common histologic sub type. Current studies implicate the VHL gene in the development of both familial and sporadic clear cell tumors.^2^ Up to 96% of clear cells RCC are associated with 3p deletion, including somatic inactivating mutations of the VHL gene. Trisomies of 3, 7, 12, 16, 17, 20, loss of Y chromosome for hereditary papillary renal cell carcinoma and c-Met mutations are responsible for sporadic papillary renal cell carcinoma.^1^^,^^3^^-^^5^

The present study is based on assessment of presence or absence of VHL mutations using Polymerase Chain Reaction in cases of renal cell carcinoma in our population. If VHL expression is noted in majority of cases the possibility of familial inheritance can be further assessed. Strict follow up of these patients and genetic screening of their family members may be useful for the identification of families and may also be helpful for further counseling regarding avoidance of risk factors like tobacco and chemicals.

This study may also assist future molecular based therapeutic strategies along with variety of adjuvant approaches including hormonal manipulation, radiotherapy, immunotherapeutics vaccines.

The objective was to detect the expression of Von Hippel Lindau gene mutation in different morphological variants of renal cell carcinoma in radical nephrectomy specimens using polymerase chain reaction (PCR) technique.

## METHODS

The present study is based on the detection of VHL gene mutations using polymerase chain reaction on radical nephrectomy specimens received in pathology department BMSI, JPMC from 1^st^ January 2007 to 31^st^ December 2012.

A total of 30 finally diagnosed cases of RCC were retrieved and eventually subjected to morphological review and PCR technique. Morphological review also included capsular invasion, vascular invasion, necrosis and perinephric fat infiltration. Materials included the following:

1. Buffered formaline fixed specimen for prospective case.
2. Paraffin embedded blocks for retrospective case.
3. Surgical pathology records.
4. Haematoxylin and Eosin stained slides for all cases.
5. Special stain PAS (periodic acid Schiff) and Hale’s colloidal Iron applied where required.
6. DNA extraction taken from paraffin embedded blocks.

***Inclusion criteria: ***All properly fixed radical nephrectomy specimens.

***Exclusion criteria: ***Small renal bioposy specimens that did not provide information regarding capsular, vascular and perinephric fat infiltration.

***Methods:***

1. For prospective cases, clinical history and relevant data was collected on a request form and recorded on proforma and Haemotoxylin and Eosin (H&E) Staining was done for all cases, PAS staining and Hale’s colloidal Iron for few cases.
2. All the slides were studied under light microscope using scanner (4X10), low power (10X10), and high power (40X10) lenses and reviewed. Capsular and vascular invasion, necrosis, perinephric fat infiltration and lymph node status was noted.
3. PCR was done for all cases by using the following:

- Primers (forward and reverse )
- Polymerase enzyme
- Template DNA
- PCR buffer

***(a)*** ***PCR*** ***conditions: ***PCR primers used for amplification are described with following components.

- In PCR, reactions mix contained 10mM Tris/HCl, 50mM KCl, 1.5mM MgCl, 50pM of each nucleotide.
- A total of 15 μl final PCR reaction volume was used for this purpose.
- The reaction volume was composed of 0.5 micrograms of DNA template, 20 pmol of each of the primers were added in two different tubes according to their base pair, 2.5 unit Taq DNA polymerase, and 0.5 mM of each dNTP in a solution of 10 mMTris-HCl, 50 mM KCl and 1.2 mM MgCl_2_, (Kappa, USA).
- The thermal cycling regimen consisted of 1 cycle of denaturation 94^o^C for 5 minutes, then repeating these conditions at 94^o^C for 45 seconds, primer annealing at 53^o^C for 1.5 minutes and extension at 72^o^C for 1.5 minutes.
- Forty five cycles were performed with the final extension at 72^o^ C for 8 minutes.

**Table:** 

***Name***	***Sequence***	***Exon***
Sense3a	GGT CTG GAT CGC GGA GGG A	1
Asense4a	GCC CGG CCT CCA TCT CCT	
Sense5a	AGT CGG GCG CCG AGG AGT	2
Asense6a	CCG TCG AAG TTG AGC CAT AC	
Sense7a	CCC AGG TCA TCT TCT GCA AT	3
Asense8a	CTG CTG GGT CGG GCC TAA G	
Sense9	GTG GCT CTT TAA CAA CCT TTG C	4
Asense10	CCT GTA CTT ACC ACA ACA ACC TTA TC	
Sense11a	CAC TGA GGA TTT GGT TTT TGC	5
Asense	TCC AGG TCT TTC TGC ACA TTT	
Sense13a	GAC ATC GTC AGG TCG CTC TA	6
Asense14a	TCA AAA GCT GAG ATG AAA CAG TG	

***(b)DNA Extraction: ***The DNA purification from tissue was carried out by using Epicenter Kit (cat# MCD85201) and the protocol was followed accordingly. In 1.5 ml eppendroff cup 50 mg of tissues was taken from the paraffin block and 600 μl T & C lysis solution was added along with diluted 1μl of 50μg/μl Proteinase K to all the sample tubes. Mix and incubate overnight at 37^o^C. At the end of incubation all the sample tubes were placed on ice for 3-5 minutes and were cooled. 200 μl of MPC Protein Precipitation Reagent was added to 600μl of lysed sample and vigorously vortexed. Then all the samples were centrifuged at 13000 rpm for 10 minutes and supernatant was obtained. This supernatant was transferred into another tube in which 500 μl of isopropanol was added. All the tubes were inverted 30-40 times to recover the DNA from supernatant. The tubes were centrifuged for 10 minutes at 13000 rpm. The supernatant was discarded without disturbing the pellet. Then 500 μl 100% ethanol was added into pellet and was centrifuged for 7 minutes at 13000 rpm. The supernatant was discarded and tubes were placed inverted slowly without disturbing the pellet. The pellet was air dried and the pellet was resuspended in 35 μl of TBE buffer. All the samples were incubated at room temperature.

PCR was performed in a tube containing 20 μl of reaction mixture made up of the following components: 10 pmol of each primers (multiplex primers) 500 μM of four deoxynucleotides, 2.5 U of Taq polymerase (Promega), 10 x PCR buffer containing and 1.5 mM MgCl_2_. 

The thermal cycler (Master Gradient PCR System, Eppendorf AG, Germany) was programmed to first incubate the sample for 5 min for 95°C followed by 40 cycles consisting of 94°C for 45 seconds, 53°C for 50 seconds and 72°C for 1.30 minutes respectively with final extension for 7 min at 72°C. The PCR amplified products were identified by electrophoresis on a 1.5% agarose gel, stained with ethidium bromide, and evaluated under transilluminator. The sizes of PCR amplified product were estimated according to the migration pattern of a 100-bp DNA ladder (Gibco BRL Life Technologies).

***(c)GEL Electrophoresis: ***The 3 μL of gel loading buffer was added in the amplified product, mixed and then loaded on 2% agarose gel electrophoresis, carried out in TBE buffer. The results were observed and the bands were compared with positive control. The size of the PCR products were determined by comparing it with a DNA ladder, and interpretation was done.

***( d)VHL positive control cases:*** The following cases of pheochromocytoma were used as positive control:

SP No. 615-11                         pheochromocytoma

SP No. 3056-10                       pheochromocytoma

SP No. 3454-10                       pheochromocytoma

***( e)VHL Negative control cases were*** *:*

SP No. 3510-10                         normal kidney tissue

SP No. 3783-12                         chronic pylonephritis

***Statistical Analysis: ***The data feeding and analysis was done on computer package SPSS (Statistical Packages of social sciences) version 16.0. Clinical characteristics were summarized in terms of frequencies and percentages for qualitative variables (gender, age grouping, site of renal nephrectomy specimens, morphological type, VHL mutation (PCR), etc., mean ± S.D. for age in year). Statistical comparison of age according to gender, site of renal nephrectomy specimen, morphological type, VHL mutation (PCR) was performed by student ‘t’ test/ANOVA and Chisquare/Fisher test (was applied for values smaller than 5) for qualitative variables. In all statistical analysis only P-value < 0.05 is considered significant.

## RESULTS

A total number of 352 renal surgical pathology cases were registered in pathology department BMSI, JPMC from 1^st^ January 2007 to 31^st^ December 2012. Out of these 274 (77.84%) were biopsies, 48 (13.64%) nephrectomies and 30 (8.5%) radical nephrectomies done for renal neoplasm.

All the radical nephrectomy specimens diagnosed as renal cell carcinoma included clear cell renal cell carcinoma 21 (70%) cases, clear cell papillary renal cell carcinoma 03 (10%), papillary renal cell carcinoma 02 (6.7%) cases and 04 (13.3%) hybrid cases showing a mixed pattern of tumors in which three cases show a combination of clear cell with focal oncocytic pattern and papillary arrangement, and one case revealing combination of clear cell with papillary arrangement with foci of chromophobe pattern. (Table-I)

Table-II shows result of PCR for VHL gene mutations done in all 30 cases. VHL gene mutations were seen in 20 (95.23%) of clear cell RCC while a single case was negative for VHL mutation. All CCPRCC were negative for VHL mutations. Among the hybrid tumor 03 cases with foci of clear cells show VHL mutations while a single case showing combination of clear cells and chromophobe cells was negative for mutations. Both the cases of papillary RCC were positive for mutations. 

Exon 3 mutations at base pair 194 seen in 11 cases and exon 2 mutation at base pasir 150-159 was seen in 18 cases including positive control cases. None of the cases showed exon 1 mutation. Table-III shows 17 (68%) RCC cases with exon 2 mutations and 08 (32%) with exon 3 mutationas.

Table-IV shows 14 cases of CCRCC, 03 hybrid tumors with exon 2 mutations and 06 cases of CCRCC and 02 case of PRCC with exon 3 mutations. 05 control cases for VHL mutations were used, the cases of pheochromocytoma showed VHL mutations, one case of normal renal tissue was negative for mutation and single case of chronic pylonephritis with end stage kidney surprisingly positive for mutation.

**Table-I:** Distribution of various morphological types of RCC (N=30).

***Morphological types***	***No of cases***	***Percentage (%)***
CCRCC	21	70%
CCPRCC	3	10%
PRCC	2	6.7%
Hybrid tumor	4	13.3%
Chromophobe tumor	0	0
Total	30	100.0%

**Table-II:** Distribution of various morphological types of RCC according to VHL mutations (N= 30).

***Morphological***	***VHL mutations***	***VHL mutations***
***types of RCC***	***+ve (%)***	***-ve (%)***
CCRCC	20 (95.23%)	1 (4.76%)
CCPRCC	0	3 (100%)
PRCC	2 (100%)	0
Hybrid tumors	3 (75%)	1 (25%)
Chromophobe RCC	0	0
Total	25 (83.3%)	05 (16.6%)

**Table-III:** Distribution of RCC according to types of exon mutations

	***No of cases***	***%***
Exon 1	0	0
Exon 2	17	68
Exon 3	08	32

**Table-IV:** Distribution of various morphological types of RCC according to Exon 2 and Exon 3 mutations

***Morphology***	***Exon 1*** ***Mutations***	***Exon 2*** ***Mutations***	***Exon 3*** ***Mutations***	***Total no of cases***
CCRCC	0	14	06	20
PRCC	0	0	02	02
Hybrid tumors	0	03	0	03

**Figure:**
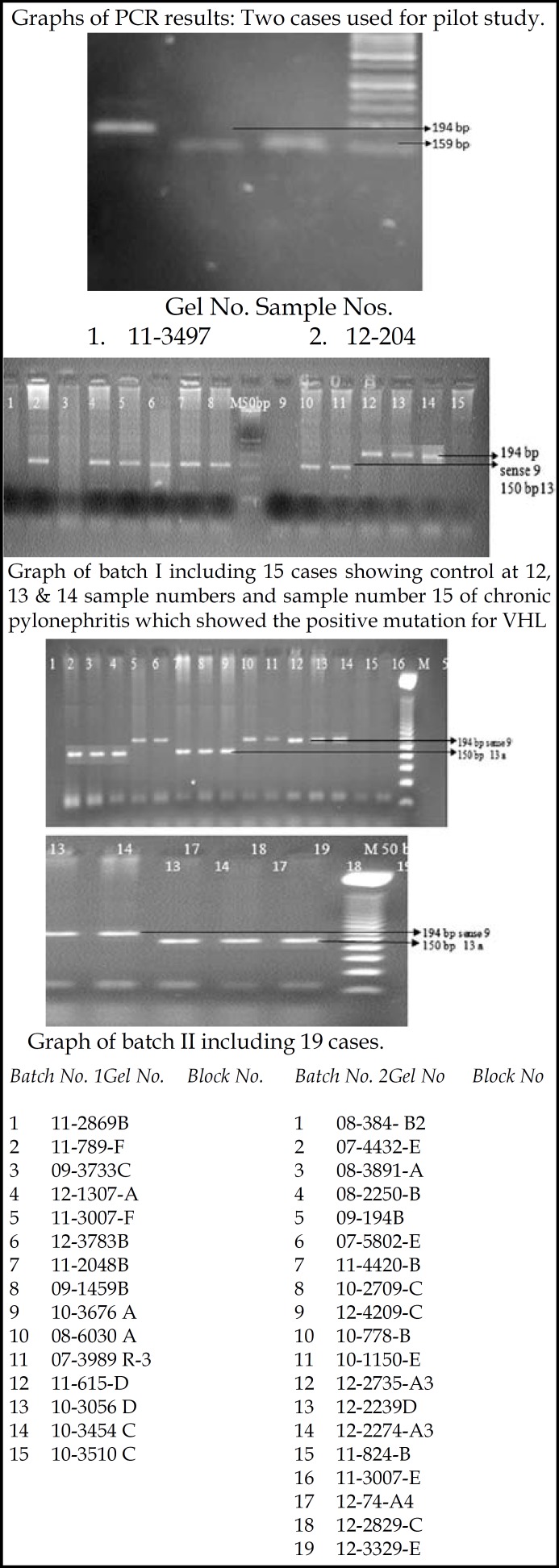


## DISCUSSION

In our study RCC was 8.5% of total renal pathologies. Agha et al.^6^ reported 12.4% of renal cell carcinoma and a study from Latif et al.^7^shows 14.36% of RCC during 1987 to 1997 in SIUT Karachi. These findings are slightly higher compared to this study probably due to a large sample size and a long study duration.

All the cases of RCC were processed for VHL gene mutations by Polymerase Chain Reaction and 83.33% cases of RCC showed VHL gene mutations. Majority of clear cell carcinoma that is 95.23% (except one case), both the cases (100%) of papillary renal cell carcinoma and 75% hybrid tumors showed VHL mutations. 

Presence of VHL gene mutations in considerable numbers of CCRCC has also been reported by other studies. Moore et al.^8^ reports 88.3% of CCRCC with VHL mutations, while Brauch et al.^9^ reports 75% and Shuin et al.(1994)^10^ reports 56% of clear cell carcinoma with VHL mutations. On the other side of spectrum, Qing et al.^11^ reports a lower figure that is of 35% of CCRCC with VHL mutations. The cases showing VHL mutation in present study showed exon 2 mutation at base pair 150 to 159 in 68% cases while 32% cases showed exon 3 mutations at base pair 194. In comparison a study by Brauch et al.^9^ reports exon 2 mutations at 119-167 base pairs in 20% cases and exon 3 mutations at 167 to 200 base pair. Qing et al.^11^, on the other hand reports exon 3 mutations in 48% and exon 2 mutations in 20% cases of CCRCC.

Feldman et al.^12^ proposed that mutations in exon 2 leads to the VHL disease phenotype. Mutations in all three exons can result in a VHL associated disease as mentioned by Kaelin and Maher^13^, Kondo.^14^ None of the cases in present study showed exon 1 mutation. Single case of CCRCC that was negative for mutations in our study could be due to possibility of other gene mutations involved such as FH, SDH, PBRM 1, and BAP 1 as expressed by Gomey and Silva^15^ and Pawloski et al.^16^

All the cases of CCPRCC were negative for VHL mutations which is in close approximation with the results of Rohan et al.^17^ showing all CCPRCC with negative results. An interesting finding in this study was that the 03 Hybrid tumors with foci of clear cells admixed with papillary and oncocytic pattern also showed the VHL mutations .Single case of hybrid tumor with large areas of chromophobe pattern was negative for mutations as in these cases other genes like BHD or FLCN mutations has been implicated as expressed in studies by Gatalcia et al^18^ and Adley et al.^19^

Surprisingly 2 cases of papillary RCC in our series showed VHL mutations with two repeated PCR analysis of these two cases. This finding warrants further investigations to evaluate the possibility of co-existence of VHL with c-Met pathway Gomey and Silva^15^ has also expressed that MET and VHL signaling pathways intersect via pVHL-mediated regulation of HIF function.

In the present study a single case of chronic pyelonephritis with end stage kidney from nephrectomy specimen of a 41 year old male patient which was processed as negative control for VHL surprisingly showed exon 3 VHL mutation. This is an incidental finding probably suggesting that the individual belonged to a family with VHL inheritance. The patient unfortunately lost follow up but provided us an important information that inherited VHL gene mutation is an existing factor in our population. As cited by Alpeers^2^, some individuals with inherited germiline VHL mutations never develop cancer as second mutation never occurs. Hence cancer may skip a generation. Keeping this in mind, the identification of VHL mutant families and subsequent follow up should not be ignored. Surveillance of families at risk and educating them regarding risk factors such as tobacco smoking, hypertension, obesity and exposure to certain chemicals may be useful. Hemminki^20^ reports welding fumes as a risk factor and lay stress on consumption of vegetables, citrus fruits and selenium as protective factors against multiple mutations of VHL in RCC.

## CONCLUSION

The present study shows that the majority of CCRCC cases showed VHL mutations supporting the possibility that VHL mutation is not uncommon in our population. Most patients were of older age group thus falling into sporadic while the younger age may have a hereditary form of RCC. In our population only exon 2 and exon 3 mutations of VHL gene were found which support the sporadic VHL mutations. The interesting findings was hybrid tumors particularly with admixture of clear cell morphology showing VHL gene mutation further confirming the role of VHL mutation in CCRCC. These hybrid tumors probably represent multiple intricate gene mutation mechanisms and require further molecular gene based studies.

## RECOMMENDATION

The Genetic counseling of concerned families particularly of young patients with positive VHL mutation is very important for awareness of the entire family. These families at risk should be selected for frequent screening program including ultrasound KUB, CT scan and MRI and should be educated and made aware regarding early detection of any important sign of kidney disease. Programmed regular strict follow up of non-functioning kidney with history of dialysis and renal cysts which are already reported as risk factors for development of RCC should be advised close follow up. Other risk factors particularly tobacco smoking, obesity, hypertension chemicals and welding fumes should be avoided. Intake of vegetables, citrus fruits and selenium are considered as protective factors against VHL mutations. Large scale study is recommended for detection of coexistence of other genes like c-Met pathway, BHD gene mutations or PBRM1 with VHL gene for the detection of causative genes for each morphological type of RCC.

## Authors Contribution:

1. ***Humera:*** Conceived, Planned, Statistical analysis and manuscript writing.
2. ***Shahnaz Imdad Kehar: ***Supervised, reviewed, helped in editing and final approval of manuscript.
3. ***Shahzad Ali:*** Data collection and clinical informations.
4. ***Naila Tariq:*** Data collection and technical help.

(All cases of renal cell were collected from archives of registered pathology specimens with permission of authorized person).
